# MapMi: automated mapping of microRNA loci

**DOI:** 10.1186/1471-2105-11-133

**Published:** 2010-03-16

**Authors:** José Afonso Guerra-Assunção, Anton J Enright

**Affiliations:** 1 European Bioinformatics Institute, Wellcome Trust Genome Campus, Hinxton, Cambridge CB10 1SD, UK; 2 PDBC, Instituto Gulbenkian de Ciência, Apartado 14, 2781-901 Oeiras, Portugal

## Abstract

**Background:**

A large effort to discover microRNAs (miRNAs) has been under way. Currently miRBase is their primary repository, providing annotations of primary sequences, precursors and probable genomic loci. In many cases miRNAs are identical or very similar between related (or in some cases more distant) species. However, miRBase focuses on those species for which miRNAs have been directly confirmed. Secondly, specific miRNAs or their loci are sometimes not annotated even in well-covered species. We sought to address this problem by developing a computational system for automated mapping of miRNAs within and across species. Given the sequence of a known miRNA in one species it is relatively straightforward to determine likely loci of that miRNA in other species. Our primary goal is not the discovery of novel miRNAs but the mapping of validated miRNAs in one species to their most likely orthologues in other species.

**Results:**

We present *MapMi*, a computational system for automated miRNA mapping across and within species. This method has a sensitivity of 92.20% and a specificity of 97.73%. Using the latest release (v14) of miRBase, we obtained 10,944 unannotated potential miRNAs when MapMi was applied to all 21 species in Ensembl Metazoa release 2 and 46 species from Ensembl release 55.

**Conclusions:**

The pipeline and an associated web-server for mapping miRNAs are freely available on http://www.ebi.ac.uk/enright-srv/MapMi/. In addition precomputed miRNA mappings of miRBase miRNAs across a large number of species are provided.

## Background

Recently, miRNAs have been shown to be a large and diverse class of regulators [1,2]. A large effort has been under way to clone and sequence miRNAs using a variety of technologies in multiple species and tissues [3,4]. These molecules are 18-22 nt and function via binding to the 3'UTRs of their target transcripts [5]. This binding event is targeted via complementarity between miRNA and target sequence. The binding of a miRNA to its target transcript causes repression of translation and also transcriptional destabilisation [6]. miRNAs have been implicated in a large and growing number of diseases and processes across both animal and plant kingdoms [7,8]. The miRBase database is the primary repository for these data [9]. It focuses on both nomenclature and recording of precursor and mature sequences and their probable genomic loci. Currently many deposited miRNAs are derived from model organisms (e.g. Human, *C. elegans*). Given that many miRNAs are highly conserved between species [10] it is likely, for example, that a miRNA discovered in *C. elegans *will also be present in *C. briggsae*. In other cases even in one species there may be multiple genomic loci which could encode a detected mature miRNA sequence and not all of these may be annotated in miRBase. This implicit bias towards model organisms hampers miRNA research in other organisms and makes evolutionary and phylogenetic analysis of miRNA families across species extremely difficult. Given a mature miRNA sequence in one species it is possible to detect the likely location of its orthologue in another species using both sequence analysis and RNA secondary structure prediction. Our assumption here is that an orthologous miRNA will possess both a high degree of similarity to the miRNA mature sequence and that identified orthologous loci should have the capability to form the stem-loop structure typical of miRNA precursors. Some groups use *ad hoc *methods for miRNA mapping analysis, however such approaches are generally either not available to the community, have not been validated or are too specific for general use. For example, miROrtho [11] provides web-access but not methods or raw data, while CoGemiR [12] provides raw data but does not allow sequence searches. Another tool, miRNAminer [13] requires the user to provide both the mature sequence and the precursor sequence and runs on a limited set of species. For these reasons, it is very difficult to directly compare the existing methods to MapMi in terms of performance. However, where possible we have compared predictions from MapMi against CoGemiR, miRNAminer and miROrtho (see Additional file 1). The most complete comparison is with miROrtho where there is a high degree of overlap between the methods, for the species where their data is freely available. When human miRBase miRNAs are used as a reference set, both methods predict a shared set of 478 loci, while miROrtho predicts 49 loci that MapMi does not and MapMi detects 139 loci not detected by miROrtho (see Additional file 1, Figure S4). Many methods have focused on prediction of novel miRNAs from genomic hairpins [14] which is a non-trivial problem. In our case we focus on the simpler task of mapping an identified miRNA in one species to others using both sequence similarity and RNA secondary structure. While our system has not been designed for predicting the loci of novel miRNAs, it is useful to leverage on the data produced by other methods, expanding it to other species. We describe our approach to miRNA mapping and demonstrate that it performs well, discriminating between true miRNAs and false-positives. The approach is freely available as both software and a web interface. Furthermore, we provide precomputed mappings of all miRBase miRNA sequences across 46 Ensembl genomes and 21 Ensembl Metazoa genomes [15]. We will maintain this resource through subsequent updates of miRBase for all species available in Ensembl.

## Implementation

### Pipeline

**Figure 1:**
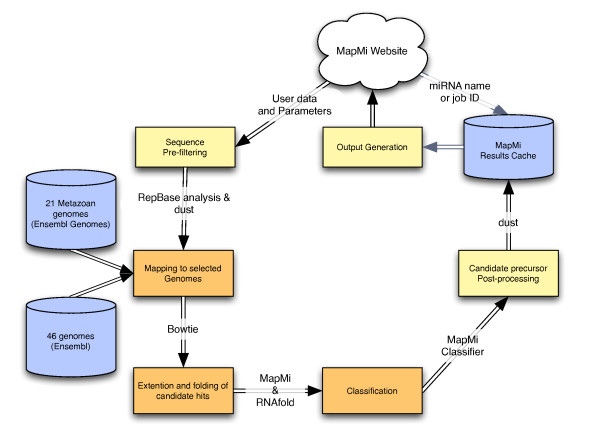
**MapMi Webserver Work flow**. Work flow of the MapMi web pipeline. The user can use the service by either providing a potential mature sequence to map, or by querying the results database, either with a miRNA name or a job id if retrieving results from a previous run.

The MapMi pipeline works as follows (Figure 1). The system is supplied with a set of input sequences corresponding to mature miRNA sequences. The user then decides which species to map these sequences against. The stand-alone version of MapMi allows the user to supply their own genomic sequence. The genomes used have previously been processed using RepeatMasker [16] to remove repetitive elements, that are not similar to known miRNAs [17] (see Additional file 1, Table S3). The provided input sequences are scanned against selected genomes using the Bowtie algorithm [18], which is designed for efficient short sequence matching. The system allows no gaps but up to three mismatches, allowing one mismatch by default. Each match is extended to produce a pair of potential miRNA precursors through extension of 110 nt (e.g. 70 nt 5' and 40 nt 3' and *vice versa*). Each of these potential precursors is then folded using *ViennaRNA *[19]. A scoring function is used to evaluate each candidate. The scoring function (see below) takes into account both the quality of the sequence match and the structure of any predicted hairpin. The best candidate is selected based on the score (either 5' or 3'). Candidates are further filtered according to a score-threshold. This is defined by the user, however a number of suggested thresholds are provided. These thresholds have been selected according to an empirical analysis of true and shuffled miRNA sequences (see Additional file 1, Table S4). All miRNA precursor loci above threshold are reported to the user with their associated scores and other relevant information. As an alternative, the user can query a database of pre-computed results, using a miRNA name as a query, and selecting the desired species and threshold.

Finally, the web version of MapMi provides detailed further analysis capabilities for precomputed results. This includes the generation and display of Maximum Likelihood phylogenetic trees (PhyML [20] & PhyloWidget [21]), Multiple sequence alignments (MUSCLE [22] & Jalview [23]) and RNA Structural logos (RNALogo [24]).

### Scoring Function

MapMi takes into account several properties of known miRNAs in its scoring function (Equation 1). In this context, Mismatches, Matches and PerfectMatches correspond to the number of nucleotides that are part of the predicted structure between the two arms of the stem loop. Mismatches correspond to the number of structurally unpaired bases, Matches to the number of structurally paired bases and PerfectMatches to actual basepairing. Mature Mismatches are obtained by parsing the output of Bowtie, the HairpinDeltaG is the value of minimum free energy returned by RNAfold, and MismatchPenalty is a parameter specified by the user. The MismatchPenalty parameter is important to distinguish sequences with mismatches from sequences with no mismatches, that can match to the same loci. The parameter can be set to a value that is large enough to enable this distinction but at the same time does not hamper the method's functionality by penalising mismatches too much (i.e. excluding sequences that have less than the maximum allowed number of mismatches, because the penalty is too high). A warning is displayed if this is likely to be the case.(1)

### Validation

The negative dataset was generated by using *ushuffle *[25] to generate 10 and 100 shuffles per initial nucleotide sequence. Due to their nucleotide composition, some of the 4,237 initial sequences could not be shuffled the required number of times. The resulting in datasets contained 42,366 and 423,343 random shuffled sequences respectively. These datasets were mapped against all 67 genomes under analysis.

### Repeat Masking

The repeat masking procedure applied to the genomes prior to the analysis is useful to avoid the detection of repeat elements that contain sequences similar to known miRNAs. Nevertheless, as a consequence of this procedure some miRBase annotated miRNAs [17] are masked and therefore reduce the sensitivity of our method (see also Additional file 1, Tables S1 and S2).

## Results and Discussion

**Figure 2:**
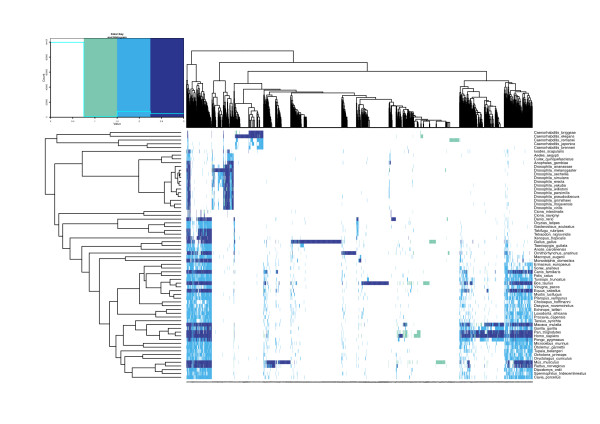
**Heatmap of Drosophilid miRNAs**. This figure was generated from a presence/absence matrix, it is color coded to illustrate the effect of mapping using MapMi in the overall view of miRNAs in the species under analysis. Dark purple corresponds to an overlap between MapMi predictions and miRBase annotation. Blue indicates miRNAs that are only present in MapMi, while green indicates miRNAs that are on miRBase but are missing from the MapMi predictions. Bias towards model organisms is readily apparent in this view. It is also clear from the image that MapMi is complementing miRBase in a way that is broadly coherent with the expected evolution of miRNAs across the metazoan lineage. The different species are ordered respecting their phylogenetic relationships, as present in the NCBI taxonomy.

**Figure 3:**
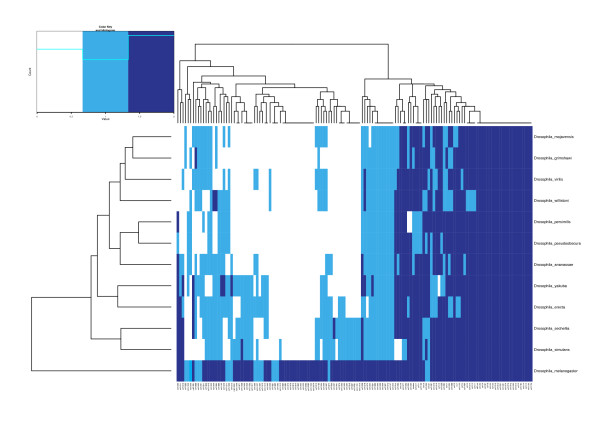
**Heatmap of Drosophilid miRNAs**. Heatmap and hierarchical clustering of miRNAs present in the 12 drosophilid genomes, as predicted by MapMi using *D. melanogaster *miRNAs as query. Dendrograms produced by clustering of the data matrix. Dark blue indicates a miRNA present both in MapMi and in miRBase, light blue indicates a miRNA present in only one of the sets.

We applied MapMi to 67 Ensembl and Ensembl Metazoa Genomes using all 7,844 metazoan miRBase miRNAs, of which 4,237 have a unique sequence (see Methods). In total, we identify 16,025 loci in all genomes under analysis using the default threshold (35), including 10,944 loci not previously reported in miRBase (Table 1). The phylogenetic profiles of miRNAs in each species are shown (Figures 2 & Additional file 1, Figure S2). The phylogeny derived from clustering these profiles broadly agrees with known phylogenetic relationships (Figure 3). Genomes were masked for repetitive elements before further analysis (see Implementation).

**Table 1:** MapMi mapping results.

Species	Loci in miRBase	Overlapping Loci (1)	New Loci (1)	Overlapping Loci (2)	New Loci (2)
*Anopheles gambiae*	67	59	27	59	12

*Bos taurus*	626	517	1002	515	187

*Caenorhabditis elegans*	174	150	96	150	3

*Canis familiaris*	325	310	251	309	89

*Ciona intestinalis*	25	21	5	21	1

*Ciona savignyi*	27	23	4	23	3

*Drosophila melanogaster*	157	129	4	129	2

*Drosophila pseudoobscura*	73	59	33	59	24

*Drosophila simulans*	70	55	50	55	47

*Equus caballus*	347	311	332	310	99

*Gallus gallus*	476	410	172	410	71

*Homo sapiens*	750	620	874	619	138

*Macaca mulatta*	483	442	730	440	166

*Monodelphis domestica*	161	146	162	145	58

*Mus musculus*	600	428	133	427	51

*Ornithorhynchus anatinus*	348	289	238	289	58

*Pan troglodytes*	604	514	751	512	149

*Rattus norvegicus*	320	297	152	297	60

*Takifugu rubripes*	133	123	124	122	95

*Xenopus tropicalis*	208	190	58	190	24

Total Loci in miRBase: 5974		Found: 5093 overlapping loci and 5232 new loci		Found: 5081 overlapping loci and 1365 new loci	

Correctly named:		5046		5035	

Overlap ratio:		(5093/5974): 85.25%		(5081/5974): 85.05%	

Correct Name Ratio:		(5046/5093): 99.07%		(5035/5081): 99.09%	

Summary of the number of loci per species that overlap miRBase annotated loci, and the number of times the overlapping loci is correctly named by MapMi. This analysis could not be performed for all species, as miRBase loci coordinates were not readily available. We present results for two different parameter sets. (1) MapMi default parameters with no repeat element post-filtering. (2) MapMi allowing only perfect matches, post-filtering for sequences that are associated with repeat elements and map to multiple places in the genome (details of filtered sequences in Additional file 1, Table S3).

### Validation

We evaluate the performance of the scoring function (see Equation 1) by comparing score distributions from a positive dataset containing 4,237 miRBase (Release 14) deposited unique sequences from Metazoan species, to a negative dataset composed of dinucleotide shuffled versions of the sequences in the positive control (see Implementation). The score distributions from both positive and negative control sequences are shown (Additional file 1, Figure S1). This illustrates that real miRNAs perform significantly better than shuffled miRNAs according to the scoring function described above. This also allows us to derive reasonable thresholds for large-scale mapping projects that balances sensitivity and specificity (Additional file 1, Table S4).

To assess the performance of our pipeline when predicting miRNA orthologues in a more general context, we analysed MapMi predictions of horse miRNAs. Horse was chosen because it was recently introduced in the latest release of miRBase. We used miRBase v13 deposited Metazoan miRNAs, that do not include horse sequences, to predict horse miRNAs. The overlap of MapMi predictions and miRBase v14 deposited horse miRNAs was 82.99% (Additional file 1, Table S5). The ability of our classifier function to distinguish miRNA hairpins from other genomic hairpins was verified by classifying the 8,494 non-miRNA hairpins as reported in [26]. We obtained a correctly classified ratio of 93.14%.

Further verification was done for the genomes for which miRBase genomic coordinates are available, to assess how many MapMi predictions overlap with miRBase annotated miRNA loci and how many of those are correctly named. We found that 85.05% of our predictions overlap with miRBase with 99.09% of those being assigned the same name as miRBase (Table 1).

Nine miRNAs appear to be highly conserved across the majority of species (Additional file 1, Table S6). These miRNAs include the well-known *let-7 *family, previously known to be highly conserved [10]. Conversely, a total of 636 miRNAs were shown to be species-specific mostly in Chicken, *C. elegans*, Cow, Platypus, Human and Mouse. This result may arise due to some organisms being more heavily profiled (e.g. Human and Mouse). Additionally, some species have few related species available for comparison (e.g. *X. tropicalis*) and would appear to have an excess of species-specific miRNAs. *Saccharomyces cerevisiae *is not believed to possess machinery for miRNA processing, however it is present in Ensembl and was retained as a negative control. As expected, no miRNAs were found in *S. cerevisiae*. These results indicate that while miRBase has excellent coverage of many species, it appears to be only capturing a fraction of the total number of miRNAs in some species. Hence, we believe that these results can complement miRBase.

## Conclusions

We present a new system for miRNA mapping through sequence similarity and secondary structure, which is available both as a stand-alone tool and an online web resource. We demonstrate the selectivity and sensitivity of the approach on a variety of datasets and have applied it to a large number of genomes. This is particularly useful for recently sequenced genomes where miRNA information may be absent or sparse. Using this approach we have mapped miRNA loci in 67 genomes, many of which are not present in miRBase. We provide a web-interface and a database of pre-computed miRNA loci, multiple sequence alignments and phylogenetic trees for many genomes and we hope this system will prove useful to the community.

## Availability and Requirements

• Project name: MapMi

• Project home page: http://www.ebi.ac.uk/enright-srv/MapMi/

• Operating system(s): Platform independent (Web-service), Linux and MacOS X (Standalone version)

• Programming language: Perl

• Other requirements: Bowtie, Dust, RNAfold (For standalone version only)

• License: GNU GPL

## Authors' contributions

JAG-A performed the analysis and wrote the manuscript. AJE conceived the experiment and also wrote the manuscript.

## Supplementary Material

Additional file 1 **Supplementary Information**. Multi-page file containing supplementary figures and tables in PDF format. Can be opened with any standard PDF viewing application (e.g. Acrobat Reader). Click here for file
