# Supplementary material for: MapMi: automated mapping of microRNA loci
Source: BMC Bioinformatics. 2010 Mar 16;11:133. doi: 10.1186/1471-2105-11-133 (PMC2858034; doi:10.1186/1471-2105-11-133)
Supplement: Additional file 1 — Supplementary Information. Multi-page file containing supplementary figures and tables in PDF format. Can be opened with any standard PDF viewing application (e.g. Acrobat Reader). [file 1471-2105-11-133-S1.PDF]

Supplementary Material for:
MapMi: Automated Mapping of miRNA loci

José Afonso Guerra-Assunção[1,2] and Anton J. Enright[1]

[1]European Bioinformatics Institute, Wellcome Trust Genome Campus,
Hinxton, Cambridge CB10 1SD, United Kingdom
[2]PDBC, Instituto Gulbenkian de Ciência, Apartado 14, 2781-901 Oeiras, Portugal

Supplementary Figures

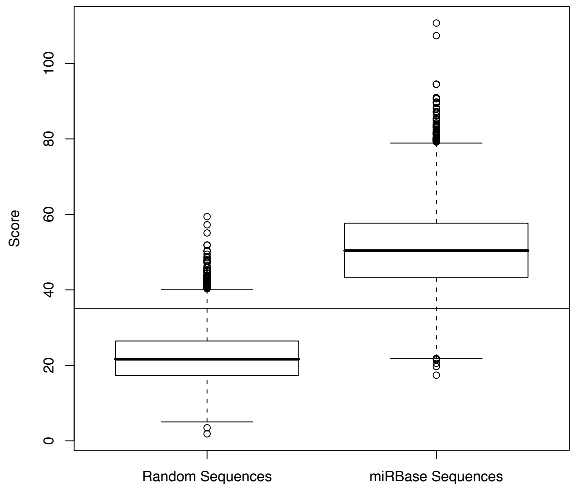

Figure S1a: Boxplot illustrating the MapMi score distribution for the 12 Drosophilids, queried with randomly di-nucleotide shuffled sequences and miRBase deposited mature sequences. The horizontal line represents the default threshold (35).

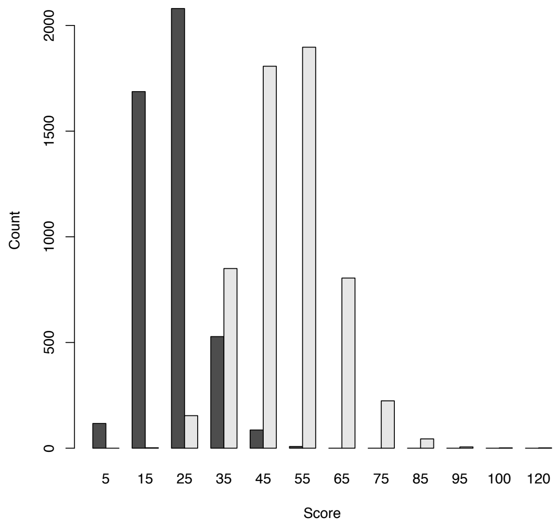

Figure S1b: Histogram showing the MapMi scores for the same run described above.

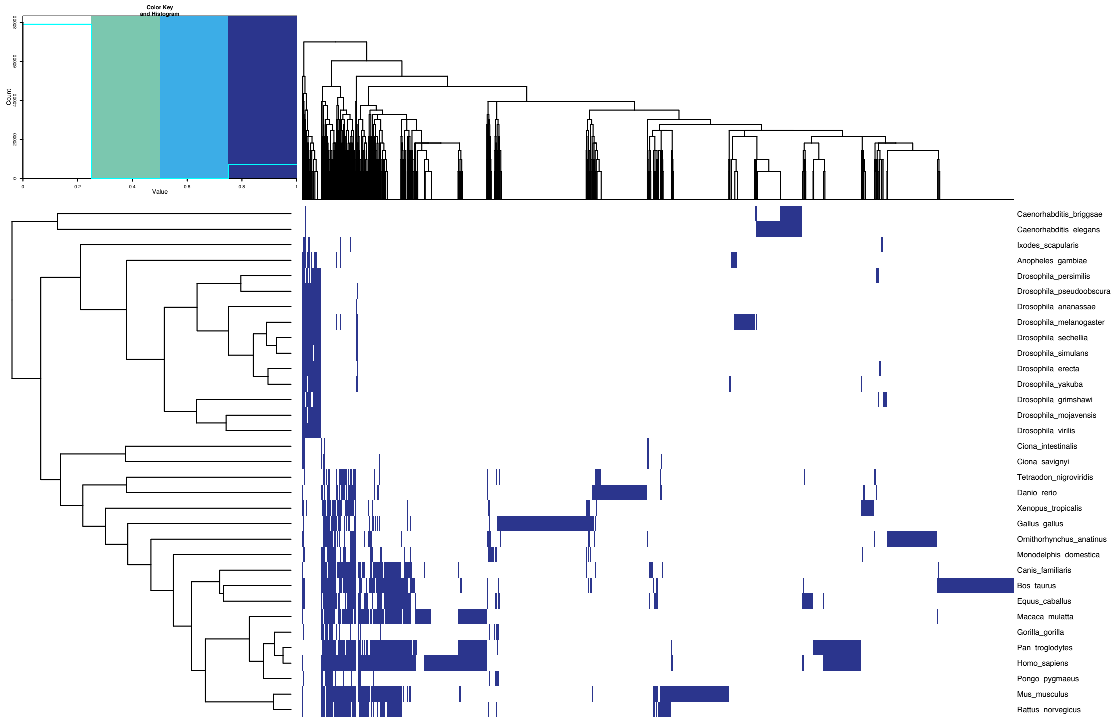

Figure S2: Heatmap containing information regarding miRNAs present in miRBase for the set of species under analysis. It was generated from a binary (presence/absence) matrix with the same parameters as Figure 2.

Supplementary Tables

Table S1: Summary of repeat elements overlapping with MapMi predicted miRNAs, for the species under analysis if no repeat filtering is used. We used the Ensembl API to assess the overlap of MapMi predicted loci with annotated repeat elements. The parameters used for this run were the default.

**Repeat Element Type**	**Frequency**
Type II Transposons	28.78%
Type I Transposons/SINE	21.70%
Type I Transposons/LINE	14.29%
Tandem repeats	10.75%
Unknown	9.79%
LTRs	7.73%
RNA repeats	3.28%
Low complexity regions	2.46%
Simple repeats	0.96%
Satellite repeats	0.18%
Other repeats	0.10%

Table S2: Summary of overlap between repeat elements and miRBase deposited miRNA loci for the species that are present in Ensembl and have miRBase coordinates available. The same procedure than Table S1 using the Ensembl API was used while producing the data for this table.

**Repeat Element Type**	**Frequency**
Type I Transposons/SINE	31.14%
Type II Transposons	28.45%
Type I Transposons/LINE	17.18%
Tandem repeats	11.57%
LTRs	5.65%
Unknown	2.13%
Simple repeats	1.74%
Low complexity regions	1.70%
RNA repeats	0.30%
Satellite repeats	0.11%
Other repeats	0.03%

Table S3: List of input miRNAs that were found to be associated with repeat elements, either because they were overrepresented in the analysis performed without repeat masking and/or matched without mismatches to one or more sequences in Repbase Update (Volume 14, Issue 8).

bmo-miR-2728	eca-miR-1302d	eca-miR-1302d	hsa-miR-548h	mmu-miR-2138	mmu-miR-709
bmo-miR-2743	eca-miR-1302e	eca-miR-1302e	hsa-miR-720	mmu-miR-2140	mmu-miR-720
bmo-miR-2747	gga-miR-1810	gga-miR-1810	mdo-miR-151	mmu-miR-2141	oan-miR-1386
bmo-miR-2749	hsa-miR-1246	hsa-miR-1246	mdo-miR-739	mmu-miR-2142	ptr-miR-1227
bmo-miR-2750	hsa-miR-1255b	hsa-miR-1255b	mml-miR-616	mmu-miR-2144	ptr-miR-1246
bmo-miR-2753	hsa-miR-1260	hsa-miR-1260	mmu-miR-1937a	mmu-miR-2146	ptr-miR-1274b
bta-miR-1814a	hsa-miR-1274a	hsa-miR-1274a	mmu-miR-1937b	mmu-miR-466f	ptr-miR-1302
bta-miR-544b	hsa-miR-1274b	hsa-miR-1274b	mmu-miR-2132	mmu-miR-467g	ptr-miR-548f
cfa-miR-1271	hsa-miR-1975	hsa-miR-1975	mmu-miR-2133	mmu-miR-690	ptr-miR-720
eca-miR-1302	hsa-miR-548f	hsa-miR-548f	mmu-miR-2135	mmu-miR-706	

Table S4: Summary of values of specificity and sensitivity of the method for each threshold. Default threshold (35) is in bold. Random di-nucleotide shuffled versions for each of the 4237 unique miRBase deposited metazoan miRNA mature sequences mapped against the 67 genomes under study and miRBase deposited precursors were used as negative and positive datasets respectively (see main text for details).

Threshold	Specificity 10 Shuffles	Specificity 100 Shuffles	Sensitivity
25	89.08%	88.04%	99.23%
26	90.68%	89.74%	98.97%
27	92.07%	91.22%	98.61%
28	93.28%	92.53%	98.23%
29	94.32%	93.65%	97.76%
30	95.22%	94.62%	97.30%
31	95.99%	95.46%	96.56%
32	96.64%	96.17%	95.66%
33	97.19%	96.78%	94.68%
34	97.66%	97.30%	93.61%
**35**	**98.05%**	**97.73%**	**92.20%**
36	98.38%	98.11%	90.67%
37	98.66%	98.42%	88.98%
38	98.89%	98.69%	86.98%
39	99.08%	98.92%	84.78%
40	99.24%	99.10%	82.64%
41	99.37%	99.25%	80.63%
42	99.48%	99.38%	78.56%
43	99.57%	99.49%	76.08%
44	99.64%	99.57%	72.64%
45	99.71%	99.65%	69.34%

Table S5: The set of identified miRNA sequences in horse (*Equus caballus*) was recently introduced in miRBase in release 14. This table illustrates the predictive power of MapMi for finding horse miRNAs, and the importance of allowing mismatches to find orthologues when no annotation is present.

Query miRBase version	Allowed Mismatches	MapMi found miRNAs	Percent Overlap with miRBase 14
13	0	271	78.09%
13	1	288	82.99%
13	2	291	83.86%
14	0	314	90.48%
14	1	314	90.48%

Table S6: This table presents the total number of miRNAs that are present in a large number of species from those under analysis (Present at least in 55 out of 67). In here the presence or absence refers to the presence of at least one orthologue of the specific miRNA in a species, not taking into account conservation of the number of loci.

	**let-7**	**miR-1**	**miR-124**	**miR-125**	**miR-133**	**miR-219**	**miR-34**	**miR-7**	**miR-92**
Present on miRBase only	3	3	1	0	1	4	2	3	0
Predicted by MapMi only	34	30	29	27	26	22	31	28	28
Present in MapMi and miRBase	28	28	34	29	32	29	29	29	32
**Total times the miRNA is present**	**65**	**61**	**64**	**56**	**59**	**55**	**62**	**60**	**60**

Comparison with other methods

CoGemiR :

It is impossible to directly compare MapMi with the CoGemiR database because loci location data is not readily available. It is true that MySQL dumps are provided, but there is no way to decode the tables back to a simple loci location table. In the supplementary information for their manuscript it is possible to see a list of miRNAs they predicted. We compared this list to the list of miRNAs predicted by MapMi (regardless of loci location) and from the 188 predictions, we fail to predict 6 miRNAs, listed in the table below.

Table S7: Elements only in "CoGemiR"

ete-mir-107	laf-mir-363	dno-mir-454
dno-mir-140	oan-mir-363	oan-mir-490

miRNAminer:

The comparison with miRNAminer was done by downloading the latest version of available predictions from their website. Nevertheless, these predictions are based on an old version of Ensembl (v48) and thus it is not guaranteed that the coordinates are maintained in the new genome assemblies on which MapMi is based. This likely accounts for a large proportion of loci that appear here to be miRNAminer specific. Since miRNAminer predictions excluded miRBase deposited miRNAs by design, it is impossible to compare miRBase overlap between these two methods.

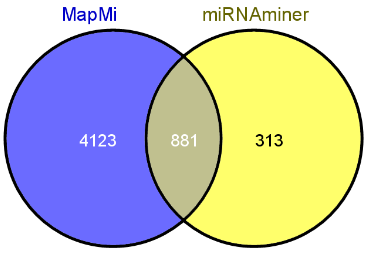

Figure S3: Venn diagram of loci overlap between MapMi and miRNAminer predicted miRNA loci.

miROrtho:

It is currently impossible to do a full direct comparison with miROrtho, as their dataset is not available for download. They only provide the users with UCSC tracks for three of the species. We downloaded these files and used them to compute the overlap between their predictions, MapMi predictions and miRBase deposited miRNAs. Results for these comparisons are shown in the Venn diagrams below. As above, venn diagrams were built using Venny [http://bioinfogp.cnb.csic.es/tools/venny/index.html].

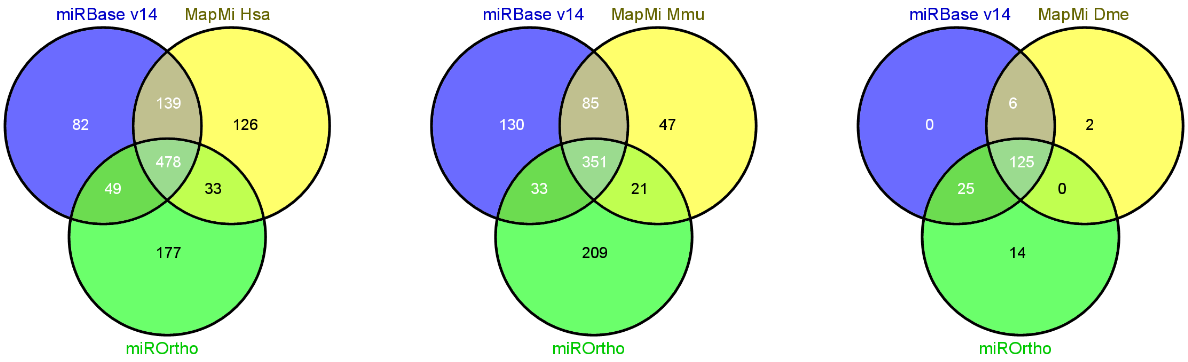

Figure S4: Venn diagrams summarizing the loci overlap between MapMi and miROrtho predictions with miRBase v14 for the three species where miROrtho data is available for download.
